# The Nightingale School and St. Thomas's Hospital —III

**Published:** 1917-11-24

**Authors:** 


### THE MATRONS' AND SISTERS' DEPARTMENT.

### THE NIGHTINGALE SCHOOL AND ST. THOMAS'S HOSPITAL—III. (*Concluded.*)

### The Second-Year and Third-Year Nurses.

After a year in the Nightingale Home "those who have passed through the course of instruction and training to the satisfaction of the committee and the hospital authorities" are taken on to the nursing staff of the hospital as extra nurses, at a salary of £22 a year, which rises in the third year, when they become staff nurses, to £24. The more serious part of their theoretical training now commences, and we admire the plan which postpones this till they have become familiar with hospital routine, for it is certain that lectures attended in the earlier stages of training are too often forgotten by the end of it. Lectures in anatomy, physiology, and chemistry are given, by members of the staff, to the second-year nurses. Classes are held by the tutor, where necessary, in elaboration of the lectures to ensure that they have been understood. Approximately in October, January, and February the nurse is examined in the above subjects, the examination consisting of a written paper and a *viva voce*.

In their third year nurses attend senior surgical (nursing) and senior medical (nursing) lectures, together with a course on gynæcological nursing. These are again followed by examinations.

### System of Examinations.

Each nurse on entering the hospital receives an examination number which she retains throughout her training. At every examination the maximum of marks is 200, divided between the paper and the *viva voce*. Failure to obtain 40 per cent., or eighty marks in all, exposes the probationer to the risk of dismissal. Nurses who gain 40 per cent. of the marks are placed in Class C; those who gain 50 per cent. in Class B; those who gain at least 60 per cent. in Class A. For the purpose of awarding medals these marks are cumulative. Through the three years' training 500 marks are awarded by the matron for good conduct and general efficiency, and to the three candidates at the head of the list when the examination and matron's marks are put together, the Gold, Silver, and Bronze Medals are awarded. At least 70 per cent. (350) of the good conduct marks must be obtained to qualify for a medal, however excellent may be the examination results.

As a means of keeping the zest and interest of nurses alive throughout their whole training this system appears to be well thought out. The results, arranged in Classes A, B and C, are published after each examination, and the final result is not left entirely dependent on the fitness of the candidate's faculties at one supreme test.

### Special Courses.

On completion of all lectures and examinations and with special permission, nurses may enter for a course in midwifery, fulfilling the requirements of the Central Midwives Board, and they are prepared for the examination by that Board.

Nurses may, on completing their hospital engagement, take a course in massage and remedial physical exercises, by which they may qualify for the examination of the Incorporated Society of Trained Masseuses. This department offers unique opportunities for obtaining thorough training under a highly-qualified sister in this branch of work. The first six months' instruction is devoted to massage, and a further six months to remedial exercises. The students see a wide variety of cases and have abundant practice, while the theoretical part of the training is exceptionally thorough.

### General Efficiency.

An admirable system has been introduced. A table of nursing points is handed to the probationer at the beginning of her course. It contains the following recommendation:—

"Will the sister, as she teaches the nursing points mentioned in the left-hand column, kindly mark the same in the column descriptive of her ward—surgical, medical, etc.? One stroke (/) to signify that the probationer has been shown, but is not perfected. A cross (×) to mean the probationer has been taught, and is quite proficient in that point. The sister placing a × will insert her initials in the right-hand column. This paper must be deposited in the matron's office by the nurse at the conclusion of her work in each ward."

Under this system an attempt is made to ensure not merely that every probationer has been shown what to do in every branch of work, but that she actually understands her business. When the ceaseless coming and going of nurses from one ward to another is remembered, together with the enormous number of small details in which perfection of method is essential, the difficulty of ensuring that each nurse receives individual and sufficient instruction in every point can hardly be exaggerated.

### The Future of the Nightingale Nurse.

The aim at St. Thomas's is not so much to prepare nurses for the lucrative calling of private work as to give them a poise and mastery over their subjects which shall fit them for posts of responsibility in institutions or in the public service. There are hundreds of matrons capable in every direction save that of lecturing to their probationers. There is room in every large hospital for a sister who shall have entire charge, under the matron, of the teaching, and nursing tutors, who are nurses first and practised teachers in addition, are likely to become more and more in request as time goes on. That St. Thomas's is well fitted to train such tutors is evident, and we await with interest the developments contemplated by the committee of the Nightingale Fund.

☛ Previous articles appeared in "The Hospital" of November 10 and 17, 1917.

